# ChatGPT in medical education: a cross-sectional analysis of usage, attitudes, perceptions, and practices among Saudi medical students

**DOI:** 10.3389/frai.2026.1751367

**Published:** 2026-04-22

**Authors:** Ali Qasem Mohammed AlAlwan, Ayoob Lone

**Affiliations:** 1 College of Medicine, King Faisal University, Al-Ahsa, Saudi Arabia; 2 Department of Clinical Neurosciences, College of Medicine, King Faisal University, Al-Ahsa, Saudi Arabia

**Keywords:** artificial intelligence, ChatGPT, attitude perception, practice, medical education, Saudi students

## Abstract

**Background:**

Generative artificial intelligence (AI) platforms such as ChatGPT are rapidly reshaping higher education, particularly in medical learning environments. Although several researches have examined students’ perceptions and attitudes toward ChatGPT in Saudi Arabia, context-specific evidence focusing on medical students and their practical engagement with ChatGPT remained limited.

**Objectives:**

This study aimed to describe patterns of ChatGPT usage, students’ perceptions, attitudes, and practices among medical students and to explore their associations with demographic and academic characteristics.

**Methods:**

A cross-sectional study was carried out between August to October 2025 involving 328 undergraduate medical students in King Faisal University, Saudi Arabia. Data were obtained using a validated 44-item instrument measuring students’ perceptions, attitudes, and practices toward ChatGPT. Descriptive and inferential statistics were performed using Statistical Package for Social Science (SPSS, Version, 27), with statistical significant set at *p* < 0.05. Parametric assumptions were tested, and multiple regression analysis was conducted to identify independent predictors.

**Results:**

An exceptional high proportion of students (97.6%) reported prior use of ChatGPT, indicating widespread familiarity with the tool. In terms of usage pattern, the majority (60.1%) indicated frequent use, followed by 26.8% who used it always, 11.3% sometimes, and only 1.08% who had never used it. Overall perception (*M* = 3.90 ± 0.69), attitude (*M* = 3.71 ± 0.62), and practice (*M* = 3.68 ± 0.73) scores reflected generally positive views. A large majority (81.7%) found ChatGPT helpful or extremely helpful in understanding medical concepts, and 73.8% believed it useful for summarizing research articles. Nonetheless, concerns persisted, with 65.2% expressed apprehension about over-reliance on ChatGPT potentially affecting originality and critical thinking. Multiple regression analyses indicated that gender, age, academic year, family status, family income, type of stay, and GPA were significant predictors of perception, attitude, and practice scores (*R*^2^ = 0.11–0.15, *p* < 0.05), with academic year, age, GPA, and gender being the most consistent predictors. Younger students (<21 years), early-year students, those with higher GPAs (>4.5), and students from higher-income families exhibited more favorable responses.

**Conclusion:**

The findings highlight strong awareness, frequent use, and positive perception toward ChatGPT among medical students at King Faisal University, viewed as an accessible and effective academic aid. Nevertheless, apprehension remain regarding reliability, ethical use, and risk of overdependence. Institutional frameworks incorporating AI literacy, critical appraisal training, and ethical guidelines are essential to ensure effective integration of ChatGPT in medical education.

## Introduction

Artificial intelligence (AI) has rapidly evolved into a transformative innovation within higher education, fundamentally reshaping the acquisition, delivery, and use of knowledge through technology-enhanced and self-directed learning environments (Grassini, 2023; Schleiss et al., 2023; Ilieva et al., 2023; George and Wooden, 2023). Within contemporary educational frameworks, digital tools increasingly support learner autonomy, independent knowledge acquisition, and personalized academic support. Among the various AI-driven technologies, large language models (LLMs) such as OpenAI’s ChatGPT have attracted substantial global attention since their release in November 2022 (OpenAI, 2023; Sallam et al., 2023a,b,c; Giansanti, 2023). These models are designed to generate coherent, human like content, provide contextually relevant answers, and support a wide range of academic activities including summarizing, problem-solving, and content creation (Syed et al., 2023; Syed et al., 2024; de Angelis et al., 2023). Within medical education, such capabilities have considerable potential to facilitate self-directed learning, enhance productivity, and improve access to knowledge beyond traditional educational resources (McCoy et al., 2024; Fuller et al., 2024).

Adoption of ChatGPT among medical students has accelerated rapidly (Eysenbach, 2023), yet it remains characterized by ambivalence. Many students’ acknowledge that ChatGPT helps streamline their workload by saving time, clarifying challenging concepts, and enhancing the effectiveness of exam preparation, assignment, and research activities (Lo, 2023; Zhang and Tur, 2023). Conversely, substantial concerns have been raised regarding factual inaccuracies, fabrication of references, threats to academic integrity, and the potential for over-reliance on AI to hinder the development of critical thinking and problem-solving skills (Tlili et al., 2023; Wise et al., 2024). These mixed perceptions reflect the dual nature of ChatGPT in education—as both a facilitator of learning and a source of emerging pedagogical and ethical challenges.

In the Kingdom of Saudi Arabia, where higher education is undergoing rapid digital transformation as part of National Vision 2030 (Elhajji et al., 2020), there is growing interest in applying AI within medical education (Almarzouki et al., 2025; Alwadani et al., 2024; Almalki et al., 2025). However, students’ readiness remains variable. National survey indicate that although Saudi medical students demonstrate moderate preparedness for AI integration, even though less than 15% had received formal AI training, while approximately one-third have participated in extracurricular AI related learning activity (Al Shahrani et al., 2024). Similarly, multi-institutional studies report limited structured exposure to AI within medical curricula, alongside insufficient institutional guidelines and practical training opportunities (Almarzouki et al., 2025; Alfahl, 2025; Gong et al., 2019). These findings highlight a gap between rapid technological advancement and systematic educational implementation.

Research focusing specifically on ChatGPT within Saudi context reflects a dual trend of excitement and concern (Alammari et al., 2025; Abouammoh et al., 2025). Several studies have explored both the benefits and potential challenges of ChatGPT across different context. For instance, Al-Sofi (2024) highlighted ChatGPT’s effectiveness in enhancing students’ academic writing skills throughout the entire writing process—from brainstorming, idea generation, and outlining to proofreading and editing assignments. However, ethical and pedagogical risks remain prominent, including plagiarism, misinformation, biased outputs, improper referencing, reduced creativity, and challenges in assessing AI-generated work (Imran and Almusharraf, 2023; AlAfnan et al., 2023; Cotton et al., 2023). Qualitative evidence further suggests cautious optimism among medical students and faculty, recognizing ChatGPT’s efficiency while expressing concerns about its potential impact on learning quality and academic integrity (Abouammoh et al., 2025).

Although a growing body of international research has examined students’ awareness and use of ChatGPT (Arum et al., 2025; Duan et al., 2025; Faraon et al., 2025; Zhang et al., 2024; Blahopoulou and Ortiz-Bonnin, 2025), and several studies have explored perceptions, attitudes, and practices among medical and healthcare students (Alammari et al., 2025; Elhassan et al., 2025; Abdelhafiz et al., 2025; Alharbi et al., 2024; Ahmed et al., 2025; Ghaffar et al., 2025), substantial gaps in the literature remain. While findings generally indicated that medical students’ view ChatGPT positively as a supportive learning tool, most research is limited by small samples and focuses primarily on awareness rather than practice, and provides little insight into the demographic or academic factors influencing engagement.

To address these gaps, the present study provides a comprehensive cross-sectional assessment of medical students’ usage patterns, perceptions, attitudes, and practices related to ChatGPT, while exploring the influence of demographic and academic characteristics on adoption and engagement. This analytical approach yields a deeper and more detailed understanding of how students engage with ChatGPT in the Saudi context and generate evidence that can guide curriculum development, policymaking and the responsible integration of AI tools into the medical education.

## Methods

### Study design

An institutional-based cross-sectional study was carried out between August and October 2025 at King Faisal University, Al-Ahsa, Saudi Arabia. The research protocol obtained ethical clearance from the Deanship of Scientific Research at King Faisal University (Approval No. KFU-REC-2025-APR- ETHICS3257). All research procedures adhered strictly to the ethical standards of the Declaration of Helsinki and other international guidelines governing research involving human participants (World Medical Association, 2013). Prior to data collection, potential participants were clearly informed about the study’s objectives, procedures, and the assurance of confidentiality. Their participation was entirely voluntary, and written informed consent was secured from all respondents. Data were collected only after confirming full compliance with all ethical and procedural requirements.

### Study sample

The students enrolled in college of medicine, King Faisal University located in eastern Governorate of Saudi Arabia were included in our research. Approximately 1,639 medical students were enrolled in this university. In Saudi Arabia, the six-year bachelor of medicine and bachelor of surgery (MBBS) program consists of three preclinical years and three clinical years including internship. The College of Medicine uses an innovative teaching style in order to fulfill its vision and goal of educating future physicians. The inclusion criteria were MBBS students, age 18 years and above, Saudi citizens. The study excluded those younger than 18 years of age, non-Saudi, medical students from private colleges, non-medical students, unwillingness to fill the questionnaire, and those who did not consent to participate.

### Sample size

The participants were recruited using a non-probability convenience sampling technique. The sample size for this study was calculated using Slovin’s formula (Slovin, 1960) with a population size of 293 participants from a recent study (Elhassan et al., 2025) with a confidence interval of 95% and margin of error of 0.05. The minimum required sample size for this study was calculated to be 321 participants. To accommodate potential non-responses, an additional seven participants were added, resulting in a total size of 328 students. The proportion of missing or incomplete response was minimum (approximately 2%) as the data was collected through personal contacts rather than an online survey.

### Data collection tools

To achieve the objectives of this study, we used previously validated and reliable tool reported in earlier studies (Sallam et al., 2023a,b,c; Caratiquit and Caratiquit, 2023). The questionnaire comprised of 44 items divided in four sections. The first section of the questionnaire captured key socio-demographic information, including gender, age, academic years, family status, and family income. Additionally, participants were asked to provide their GPA for the previous semester, measured on a scale out of 5, with 1 representing a GPA between 4.5 and 5.0, and 5 representing a GPA below 2.0. This 5-point scale was employed to enable a standardized assessment of academic performance across participants. In addition, two preliminary items were included into this section to evaluate participants’ prior exposure to ChatGPT and the frequency of its use. Prior exposure was recorded using a binary Yes/No format, while frequency of use was measured on a 4-point Likert scale, ranging from 4 (Always) to 1 (Never). The second section of the questionnaire assessed participant’s perceptions using three items, examining the extent to which respondents found ChatGPT helpful for understanding medical concepts, its perceived accuracy, and its usefulness in summarizing research articles. Responses were rated on a 5-point Likert scale, ranging from 5 (Extremely helpful) to 1 (Not at all helpful). The third section explored participants attitude toward ChatGPT, comprises of 18 statements measured on a 5-point Likert scale (5 = Strongly agree, 1 = Strongly disagree). This section aimed to capture students’ options regarding the reliability of ChatGPT’s information, potential risks of over-dependence, and its effects on fostering critical thinking skills among students. The fourth section focused on practices, consists of 13 items, also evaluated using a 5-point Likert scale (5 = Strongly agree, 1 = Strongly disagree). This section investigated practical engagement with ChatGPT, including its role in enhancing efficiency when seeking information and participants’ likelihood of recommending the tool to peers to support their academic activities. Overall, the questionnaire demonstrated moderate to high internal consistency across all domains, supporting its suitability for assessing perceptions, attitudes, and practices related to ChatGPT among Saudi medical students.

### Procedure

After obtained the necessary approval from the college administration, the student representatives of the college were contacted to facilitate participant’s recruitment. Each student batch maintained a dedicated WattsApp group for academic communication and the administrators of these groups were requested to disseminate information about the study with their respective members. Email addresses were then collected from students who expressed willingness to participate. Subsequently, the participants were contacted individually by trained senior medical students responsible for data collection. Prior to data collection, the objectives of the study were clearly explained to all participants, and informed consent was obtained. Participants received detailed instructions on how to complete the questionnaire and were assured of the confidentiality and anonymity of their responses. They were also informed that participation was entirely voluntary. Completing the questionnaire required approximately 10–15 min, after which participants were sincerely thanked for their time and cooperation.

### Statistical analysis

After data collection, a comprehensive data cleaning process was undertaken. The questionnaire responses were evaluated based on the following predefined exclusion criteria. Responses containing excessive missing data, defined as two-third or more of the total items left unanswered, responses demonstrating uniform or highly repetitive answer patterns and questionnaire completed in less than 5 min. These time thresholds were established as reasonable completion durations based on pilot study conducted on 25 medical students for this study.

Data were then carefully validated and transferred to the Statistical Package for the Social Sciences (SPSS) software (Version, 27, Chicago, IL, United States) for analysis. Graphs were created using Microsoft Excel. All statistical analyses were performed using two–tailed tests with an alpha (*α*) level of 0.05, and a *p*-value <0.05 was considered statistically significant. Descriptive statistics were used to summarize the data, with socio-demographic variables presented as frequencies and percentages, and numerical variables expressed as mean and standard deviation. Likert-scale–derived composite domain scores were treated as continuous variables, consistent with common practice in educational and behavioral research when aggregated scores approximate normal distributions. The association between demographic variables and perception, attitude, and practice scores was examined using appropriate parametric and non-parametric tests. Independent-samples t-tests were applied for binary variables (e.g., gender, age and family status). For variables with more than two categories, including academic year, family income, and GPA categories, one-way analysis of variance (ANOVA) was used. Where normality assumptions were violated, the Kruskal–Wallis test was applied as a non-parametric alternative, with *post hoc* analyses conducted when appropriate. The mean score for each domain was computed based on the 5-ponitLikert scale and results were summarized using the mean and SD. Percent scores for the three domains were further categorized into levels based on the mean: scores below the mean were classified as ‘fair’ while scores above the mean were classified as ‘good.’ To identify the independent predictors of perception, attitude, and practice scores, multiple linear regression analyses were conducted. Demographic variables including gender, age, academic year, family status, area of residence, family income, type of stay, and GPA were entered simultaneously as predictor variables. Regression assumptions, including linearity, normality, and multicollinearity, were checked prior to analysis. Regression coefficients (unstandardized B and standardized *β*), 95% confidence intervals, and significance levels were reported for all models. The proportion of variance explained by each model was assessed using *R*^2^, and the overall model fit was evaluated using the *F*-test.

## Results

### Participant characteristics

Table 1 provides a comprehensive overview of the demographic characteristics of the study participants. A selected sample comprised of 328 undergraduate medical students enrolled in College of Medicine, King Faisal University, Al-Ahsa, Saudi Arabia. Among these participants, 174 were males (53.0%) and 154 were females (47.0%). The sample was skewed toward students in the earlier academic years, with 35.4% in the first year, whereas fifth-year (16.8%) students are underrepresented.

**Table 1:** Demographic characteristics of participants.

Characteristics	*N* (328)	%
Gender		
Male	174	53.0
Female	154	47.0
Age		
<21 years	187	57.0
>21 years	141	43.0
Academic year		
1st	116	35.4
2nd	59	18.0
3rd	53	16.2
4th	45	13.7
5th	55	16.7
Family status		
Nuclear	191	58.2
Joint	137	41.8
Area of residence		
Urban	244	74.4
Rural	84	25.6
Family income (SAR)		
<5,000	31	9.5
5,001–10,000	58	17.7
10,001–15,000	75	22.8
>15,001	164	50.0
Type of stay		
Living with family	233	71.0
University Housing	78	23.8
Sharing Apartment	17	5.2
GPA in previous semester		
4.5–5	170	51.8
4–4.4	122	37.2
3–3.9	25	7.6
2–2.9	11	3.4
<2	0	0

In terms of area of residence, the majority of participants belong to urban areas (74.4%), with only 25.6% from rural areas. A substantial portion of the participants reported a family income exceeding 15001SAR (50.0%). The majority of study participants were living with their family, while the least number of participants (5.2%) were living in shared apartments. Regarding grade distribution, the majority of students (51.8%) achieved a higher GPA, while none of the student obtained a GPA below 2.0 in the previous semesters.

### Exposure of ChatGPT

**Figure 1:**
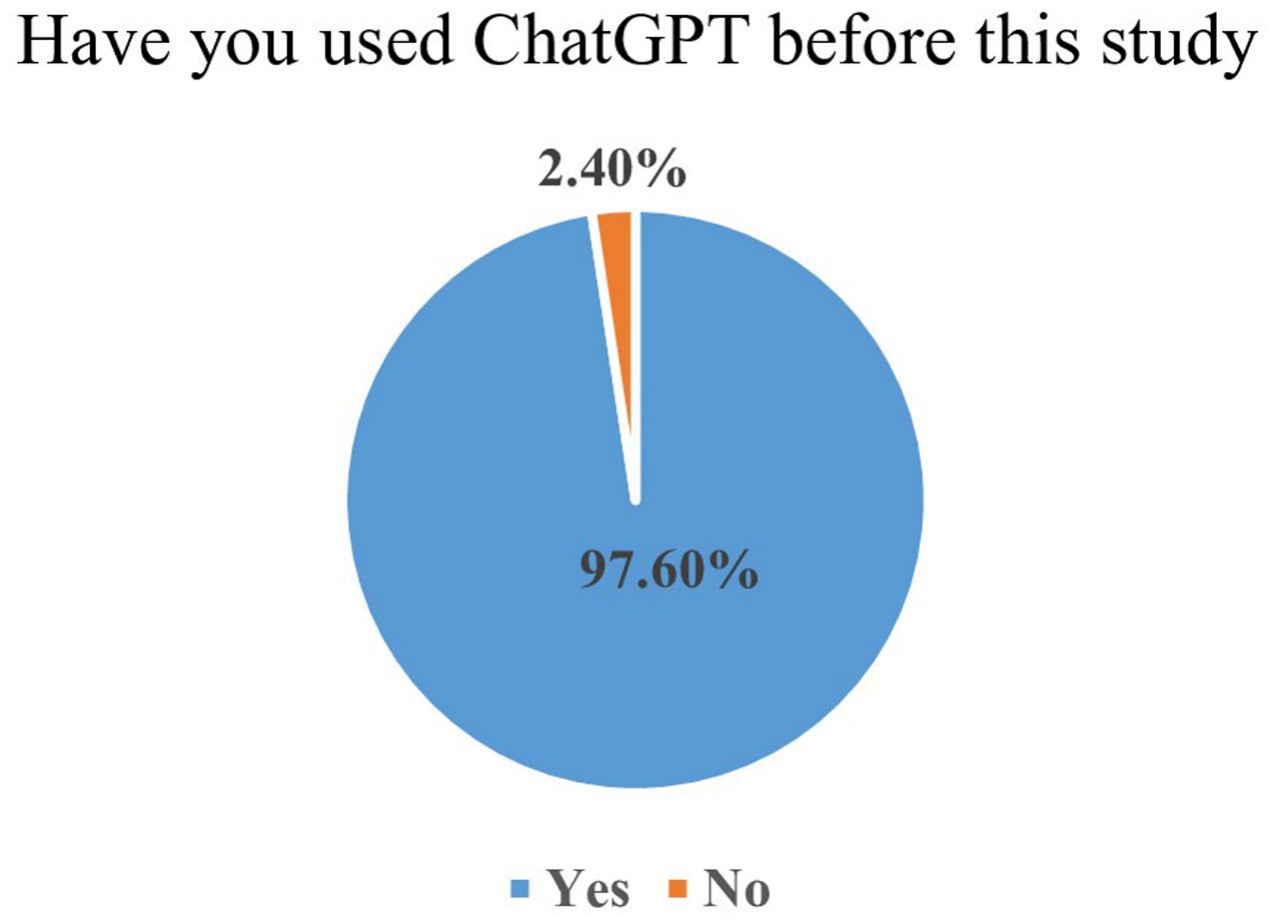
Exposure of ChatGPT among medical students.

The Figure 1 illustrated participant’s prior experience with ChatGPT. The overwhelming majority of students reported previous use of ChatGPT, while only a very small minority indicated no prior exposure. This highlights extensive awareness and prior exposure to AI-based educational tools among Saudi medical students.

### Usage of ChatGPT

**Figure 2:**
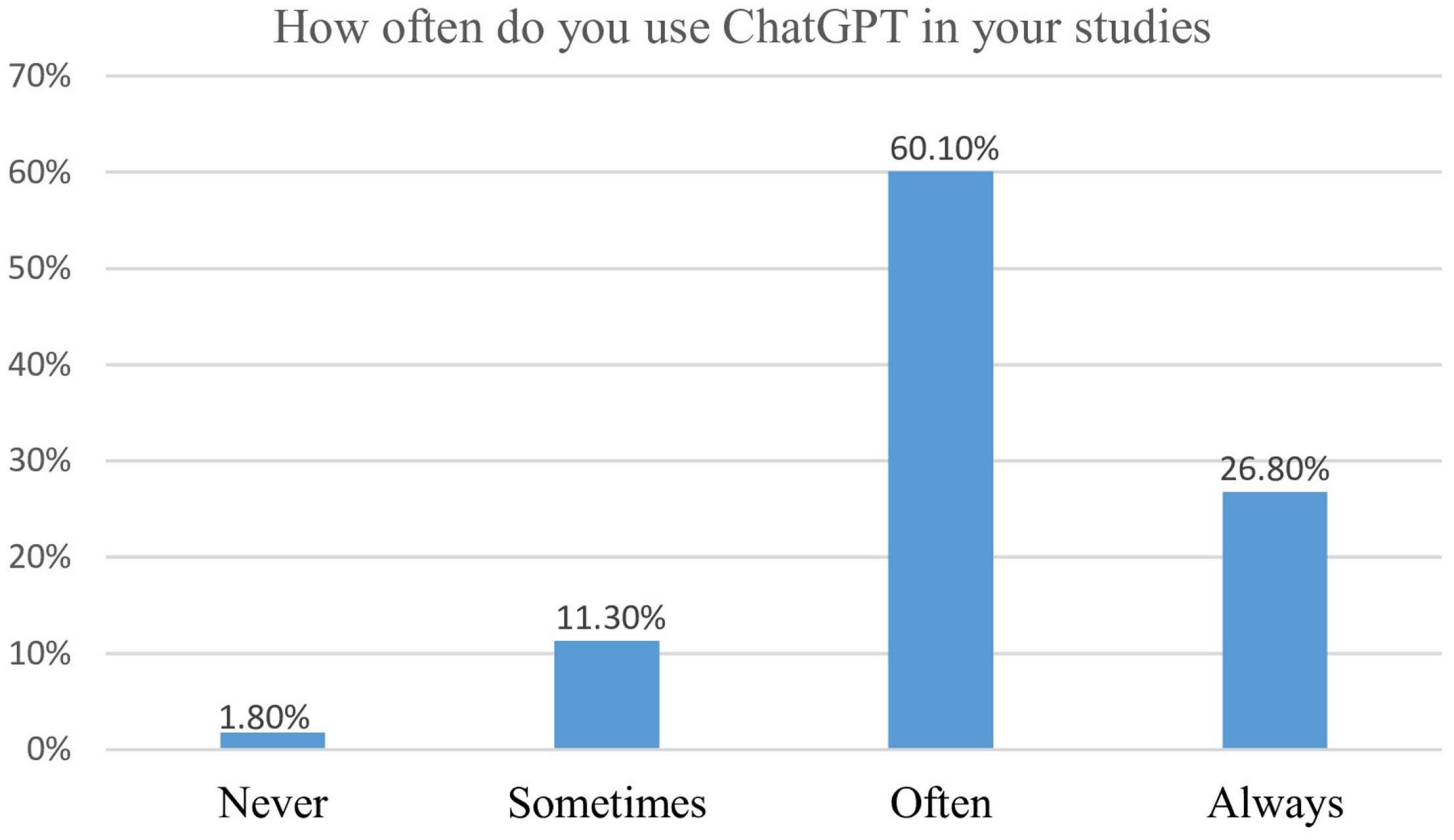
Usage of ChatGPT among medical students.

Figure 2 presents the usage pattern of ChatGPT among the study participants. Most students reported using ChatGPT either often or always, whereas a smaller proportion indicated occasional use, and only a negligible minority reported never using the tool. These findings indicate that a moderate to high level of adoption of ChatGPT, with nearly four out of five participants having some degree of experience. The predominance of the “often” and “always” categories suggests that ChatGPT is recognized as an accessible academic resource, though it has not yet achieved universal reliance. This usage pattern underscores the growing acceptance of AI-driven tools in medical education and highlights the need for structured institutional support and digital literacy initiatives.

### Perception, attitude and practice of ChatGPT

Overall, participants demonstrated generally positive perceptions, attitude, and practices toward ChatGPT. The mean scores for all three domains exceeded three on the 5-point Likert scale, reflecting a favorable overall evaluation of the tool. Nevertheless, some variability was observed across the responses, as reflected in the standard deviations. In particular, the perception domain (*M* = 3.90; SD = 0.69) demonstrated a slightly higher standard deviation compared to the attitude (*M* = 3.71; SD = 0.62) and practice domain (*M* = 3.68; SD = 0.73), suggesting a broader dispersion of opinions. This indicates that while ChatGPT is generally well-received, participant perspectives vary across domains (Table 2).

**Table 2:** Perception, attitude and practice scores toward ChatGPT among medical students.

	Mean	SD
Perception scores	3.90	0.69
Attitude scores	3.71	0.62
Practice scores	3.68	0.73

Table 3 provides a comprehensive summary of medical students’ responses to individual items assessing medical students’ perceptions, attitudes, and practices regarding ChatGPT. Overall, the findings indicate a predominantly positive perception of ChatGPT as a helpful educational resource. A large proportion of students (81.7%) found it helpful of extremely helpful for understanding medical concepts, and 73.8% considered it beneficial for summarizing research papers. While 40.2% of respondents remained neutral regarding ChatGPT’s accuracy, nearly 45.4% rated it as helpful or extremely helpful.

**Table 3:** Descriptive statistics for perception, attitude and practice responses toward ChatGPT among medical students.

	Not helpful at all *N* (%)	Not helpful *N* (%)	Neutral *N* (%)	Helpful *N* (%)	Extremely helpful *N* (%)
Perception					
How helpful do you think ChatGPT could be in understanding medical concepts?	7 (2.1)	13 (4.0)	40 (12.2)	115 (35.1)	153 (46.6)
How likely do you think it is that ChatGPT could provide accurate medical information?	6 (1.8)	41 (12.5)	132 (40.2)	100 (30.5)	49 (14.9)
ChatGPT could be a helpful tool for summarizing research papers	6 (1.8)	24 (7.3)	56 (17.1)	97 (29.6)	145 (44.2)

	Strongly disagree *N* (%)	Disagree *N* (%)	Neutral *N* (%)	Agree *N* (%)	Strongly agree *N* (%)
Attitude					
I am concerned about the reliability of the information provided by ChatGPT	10 (3.0)	11 (3.4)	100 (30.5)	142 (43.3)	65 (19.8)
I am afraid of relying too much on ChatGPT and not developing my critical thinking skills	26 (7.9)	41 (12.5)	47 (14.3)	102 (31.1)	112 (34.1)
I am concerned about the potential security risks of using ChatGPT	37 (11.3)	33 (10.1)	106 (32.3)	74 (22.6)	78 (23.8)
I am afraid of becoming too dependent on technology like ChatGPT	23 (7.0)	42 (12.8)	52 (15.9)	83 (25.3)	128 (39.0)
I am afraid that using ChatGPT would result in a lack of originality in my university assignments and duties	41 (12.5)	43 (13.1)	85 (25.9)	74 (22.6)	85 (25.9)
I am afraid that the use of the ChatGPT would be a violation of academic and university policies	38 (11.6)	31 (9.5)	74 (22.6)	110 (33.5)	75 (22.9)
I am concerned about the potential privacy risks that might be associated with using ChatGPT	45 (13.7)	57 (17.4)	92 (28.0)	57 (17.4)	77 (23.5)
I am enthusiastic about using technology such as ChatGPT for learning and research.	14 (4.3)	26 (7.9)	100 (30.5)	74 (22.6)	114 (34.8)
I believe technology such as ChatGPT is an important tool for academic success.	3 (0.9)	15 (4.6)	58 (17.7)	107 (32.6)	145 (44.2)
I think that technology like ChatGPT is attractive and fun to use.	8 (2.4)	21 (6.4)	43 (13.1)	94 (28.7)	162 (49.4)
I am always keen to learn about new technologies like ChatGPT.	12 (3.7)	39 (11.9)	60 (18.3)	87 (26.5)	130 (39.6)
I trust the opinions of my friends or colleagues about using ChatGPT.	5 (1.5)	23 (7.0)	119 (36.3)	95. (29.0)	86 (26.2)
I consider using ChatGPT for help with my studies	3 (0.9)	12 (3.7)	63 (19.2)	101 (30.8)	149 (45.4)
I am comfortable with using ChatGPT to generate content for assignments	4 (1.1)	25 (7.6)	81 (24.7)	100 (30.5)	118 (36.0)
I am concerned about the potential for misuse of ChatGPT in medical education	13 (4.0)	15 (4.6)	109 (33.2)	96 (29.3)	95 (29.0)
I am concerned with the accuracy of information ChatGPT might generate on complex medical topics	16 (4.9)	22 (6.7)	107 (32.6)	85 (25.9)	98 (29.9)
I think ChatGPT could be a reliable source for preparing medical exams	27 (8.2)	64 (19.5)	104 (31.7)	56 (17.1)	77 (23.5)
It is important for medical schools to establish clear guidelines on the use of AI tools like ChatGPT	15 (4.6)	19 (5.8)	108 (32.9)	109 (33.2)	77 (23.5)
Practice					
ChatGPT helps me to save time when searching for information	11 (3.4)	1 (0.3)	34 (10.4)	104 (31.7)	178 (54.3)
For me, ChatGPT is a reliable source of accurate information.	25 (7.6)	72 (22.0)	134 (40.9)	72 (22.0)	25 (7.6)
I recommend ChatGPT to my colleagues to facilitate their academic duties	13 (4.0)	31 (9.5)	66 (20.1)	118 (36.0)	100 (30.5)
ChatGPT is more useful than other sources of information that I have used previously.	23 (7.0)	23 (7.0)	107 (32.6)	87 (26.5)	88 (26.8)
I have used tools or techniques similar to ChatGPT in the past.	48 (14.6)	56 (17.1)	63 (19.2)	119 (36.3)	42 (12.8)
I spontaneously find myself using ChatGPT when I need information for my university assignments and duties.	16 (4.9)	25 (7.6)	91 (27.7)	88 (26.8)	108 (32.9)
I often use ChatGPT as a source of information in my university assignments and duties.	17 (5.2)	30 (9.1)	93 (28.4)	104 (31.7)	84 (25.6)
I think that relying on technology like ChatGPT can disrupt my critical thinking skills.	27 (8.2)	25 (7.6)	97 (29.6)	100 (30.5)	79 (24.1)
I appreciate the accuracy and reliability of the information provided by ChatGPT.	59 (18.0)	45 (13.7)	121 (36.9)	77 (23.5)	26 (7.9)
I believe that using ChatGPT can save time and effort in my university assignments and duties.	12 (3.7)	4 (1.2)	70 (21.3)	91 (27.7)	151 (46.0)
It does not take a long time to learn how to use ChatGPT.	12 (3.7)	5 (1.5)	42 (12.8)	111 (33.8)	158 (48.2)
ChatGPT does not require extensive technical knowledge.	14 (4.3)	13 (4.0)	65 (19.8)	98 (29.9)	138 (42.1)
I am interested in using more sophisticated AI tools to personalize my learning experience	13 (4.0)	11 (3.4)	92 (28.0)	111 (35.4)	96 (29.3)

Regarding attitudes, participants demonstrated both enthusiasm and cautious toward ChatGPT use. Most participants expressed strong interest in technology integration in medical education, with 76.8% agreeing or strongly agreeing that tools like ChatGPT are important for academic success. Similarly, 66.5% reported that they enjoy using ChatGPT and find it engaging, suggesting a favorable disposition toward technological learning aids. However, concerns were also evident: more than 65% agreed that reliance could affect critical thinking and originality, and nearly half reported privacy, ethical, and academic integrity concerns.

In practice, the majority reported integrated ChatGPT into their academic routines. Over 85% agreed it save time when searching for information, and 66.5% recommended it to peers. Approximately 75% of respondents found easy to use, requiring minimal technical expertise, which underscores its accessibility for students with varying levels of digital proficiency. However, more than half of the participants voiced concern that reliance on ChatGPT might negatively impact critical thinking, highlighting an ongoing tension between efficiency and academic development. Overall, students generally hold positive views of ChatGPT and integrate it meaningfully into their academic tasks, they simultaneously recognize the need for responsible and ethical use to avoid potential pitfalls such as overdependence, misinformation, and diminish originality.

### Perception, attitude and practice and demographic factors

Table 4 provides a detailed comparison of perception, attitude, and practice scores related to ChatGPT use among medical students. Statistically significant differences (*p* < 0.05) were observed across multiple subgroups, highlighting the influence of demographic and educational factors on students’ engagement with ChatGPT.

**Table 4:** Comparison of perception, attitude and practice scores among medical students based on demographic factors.

	Group	Mean	SD	*p*-value
Perception				
Gender	Male	3.96	0.65	0.11
	Female	3.83	0.72	
Age	<21 years	3.99	0.71	0.01*
	>21 years	3.79	0.65	
Academic Year	1st year	4.07	0.52	0.01**
	2nd year	4.20	0.64	
	3rd year	3.55	0.99	
	4th year	3.60	0.58	
	5th year	3.81	0.55	
Family status	Nuclear	3.80	0.77	0.01*
	Joint	4.05	0.53	
Area of residence	Urban	3.87	0.72	0.19
	Rural	3.98	0.57	
Family income (SAR)	<5,000	3.81	0.47	0.01**
	5,001–10,000	3.91	0.71	
	10,001–15,000	3.70	0.85	
	>15,001	4.00	0.61	
Type of stay	Living with family	3.86	0.75	0.16
	University Housing	4.03	0.53	
	Sharing Apartment	3.84	0.31	
GPA in previous semester	4.5–5	4.06	0.59	0.01**
	4–4.4	3.67	0.78	
	3–3.9	4.06	0.57	
	2–2.9	3.60	0.68	
Attitude				
Gender	Male	3.74	0.64	0.31
	Female	3.67	0.60	
Age	<21 years	3.80	0.62	0.01*
	>21 years	3.59	0.60	
Academic year	1st year	3.68	0.48	0.01**
	2nd year	4.16	0.75	
	3rd year	3.30	0.52	
	4th year	3.77	0.58	
	5th year	3.64	0.56	
Family status	Nuclear	3.79	0.70	0.01*
	Joint	3.60	0.48	
Area of residence	Urban	3.75	0.64	0.07
	Rural	3.61	0.58	
Family income (SAR)	<5,000	4.07	0.62	0.01**
	5,001–10,000	3.71	0.56	
	10,001–15,000	3.41	0.57	
	>15,001	3.78	0.62	
Type of stay	Living with family	3.70	0.64	0.01**
	University Housing	3.86	0.57	
	Sharing Apartment	3.24	0.21	
GPA in previous semester	4.5–5	3.84	0.63	0.01**
	4–4.4	3.57	0.63	
	3–3.9	3.66	0.45	
	2–2.9	3.41	0.15	
Practice				
Gender	Male	3.55	0.83	0.01*
	Female	3.82	0.55	
Age	<21 years	3.68	0.81	0.98
	>21 years	3.68	0.61	
Academic year	1st year	3.90	0.45	0.01**
	2nd year	3.36	1.21	
	3rd year	3.53	0.44	
	4th year	3.74	0.65	
	5th year	3.64	0.63	
Family status	Nuclear	3.67	0.82	0.84
	Joint	3.69	0.52	
Area of residence	Urban	3.67	0.79	0.59
	Rural	3.71	0.49	
Family income (SAR)	<5,000	2.91	1.51	0.01**
	5,001–10,000	3.73	0.56	
	10,001–15,000	3.69	0.42	
	>15,001	3.80	0.56	
Type of stay	Living with family	3.75	0.47	0.01**
	University Housing	3.57	1.20	
	Sharing Apartment	3.20	0.42	
GPA in previous semester	4.5–5	3.81	0.52	0.01**
	4–4.4	3.52	0.96	
	3–3.9	3.74	0.48	
	2–2.9	3.33	0.25	

*p < 0.01 (*t*-test), **p < 0.01 (one-way ANOVA).

For perception scores, age, academic year, family type, family income and GPA demonstrated significant associations. Younger students (<21 years) reported higher perception scores and those in early years academic years (1st and 2nd year) also reported higher perception scores, suggesting that early-stage learners consider ChatGPT as a valuable resource for mastering foundational concepts. Students from joint families and higher-income households exhibited more favorable perceptions, which may reflect greater exposure to technological resources and supportive learning environment. Similarly, students with higher GPAs (>4.5) demonstrated the most favorable perceptions, indicating that academic achievers are more inclined to view ChatGPT as a beneficial educational tool.

In terms of attitudes, significant variation were noted across several demographic and academic characteristics, including age, academic year, family type, family income, living arrangements and GPA. Younger students (<21 years) and those in the second academic year recorded the highest mean attitude scores, reflecting optimism toward integrating ChatGPT into learning. Students from nuclear families, living with their families and higher GPA groups also expressed stronger positive attitude, while those from lower-income families (<5000SAR) surprisingly showed relatively higher enthusiasm toward AI tools—possibly viewing them as affordable alternatives to private tutoring or paid learning platforms.

Regarding practice scores, gender, academic year, family income, type of stay and GPA were significant associated with ChatGPT usage. Female students, reporting higher practice scores than male counterparts. Students in their first academic year recorded the highest mean attitude scores, indicating that ChatGPT may serve as an early academic support mechanism for newly enrolled medical students as they adapt to self-directed learning expectations. Students living with their families also demonstrated significantly higher practice scores, suggesting that stable home environments may promote consistent academic technology use. Additionally, students with higher GPA and those from higher-income households again reported more active engagement with ChatGPT. These results indicate that adoption patterns are influenced by a combination of academic stage, socioeconomic status, and prior exposure to learning technologies.

A separate multiple linear regression analysis was conducted to identify the demographic predictors of perception, attitude, and practice scores regarding ChatGPT among medical students. The results, presented in Table 5, indicate that the demographic variables jointly contributed to the variance in each outcome.

**Table 5:** Result of multiple regression analysis predicting perception, attitude and practice from demographic variables (*N* = 328).

Predictor	*R*	*R* ^2^	*F*(8, 319)	Unstandardized coefficient		Standardized coefficient	Level of significance	95% CI	
				*B*	SE	*β*		Lower	Upper
Perception									
Gender	0.36	0.13	5.98, *p* < 0 0.01	−0.17	0.08	−0.13	0.03	−0.33	−0.02
Age				0.06	0.13	0.05	0.62	−0.19	0.33
Academic year				−0.11	0.04	−0.24	0.01	−0.20	−0.03
Family status				0.24	0.08	0.17	0.01	0.09	0.39
Area of residence				0.11	0.09	0.07	0.19	−0.06	0.28
Family income (SAR)				0.03	0.04	0.05	0.39	−0.04	0.11
Type of stay				0.05	0.07	0.04	0.48	−0.08	0.18
GPA in previous semester				−0.14	0.05	−0.16	0.01	−0.24	−0.04
Attitude									
Gender	0.33	0.11	4.96, *p* < 0.01	−0.10	0.07	−0.08	0.16	−0.24	0.04
Age				−0.39	0.21	−0.31	0.01	−0.63	−0.16
Academic year				0.09	0.04	0.21	0.03	0.01	0.17
Family status				−0.16	0.07	−0.13	0.02	−0.30	−0.02
Area of residence				−0.04	0.08	−0.03	0.60	−0.20	0.11
Family income (SAR)				−0.05	0.03	−0.07	0.19	−0.11	0.02
Type of stay				−0.01	0.06	−0.01	0.96	−0.12	0.17
GPA in previous semester				−0.19	0.05	−0.23	0.01	−0.28	0.10
Practice									
Gender	0.38	0.15	6.93, *p* < 0.01	0.19	0.08	0.13	0.02	0.32	0.35
Age				0.43	0.14	0.29	0.01	0.16	0.70
Academic year				−0.15	0.05	−0.32	0.01	−0.24	−0.06
Family status				−0.02	0.08	−0.01	0.82	−0.18	0.14
Area of residence				−0.03	0.09	−0.02	0.77	−0.20	0.15
Family income (SAR)				0.14	0.04	0.20	0.01	0.06	0.22
Type of stay				−0.22	0.07	−0.18	0.01	−0.36	−0.09
GPA in previous semester				−0.05	0.05	−0.06	0.31	−0.16	0.05

Gender coded as 0 = male and 1 = female; Age (<21 years = 1, >21 years = 2); Academic years (First year = 1, second year = 2, third year = 3, fourth year = 4, fifth year = 5); Family status (nuclear = 1, joint = 2); Ares of residence (Urban = 1, Rural = 2); monthly income (Saudi Riyals) (<5,000 = 1, 5,001–10,000 = 2, 10,001–15,000 = 3, >15,000 = 4); Type of stay (Living with family = 1, university housing = 2, Sharing Apartment = 3). B = unstandardized coefficient; β = standardized coefficient; SE = standard error; CI = confidence interval; *p* < 0.05 was considered statistically significant.

For perception, the regression model was significant, *R* = 0.36, *R*^2^ = 0.13, *F*(8, 319) = 5.98, *p* < 0.01, explaining 13% of the variance in perception scores. Significant predictors included gender (*β* = −0.13, *p* = 0.03), academic year (*β* = −0.24, *p* = 0.01), family status (*β* = 0.17, *p* = 0.01), and GPA in the previous semester (*β* = −0.16, *p* = 0.01), indicating that males, students in early years, those from joint families, and students with higher GPA reported different perception scores. Age, area of residence, family income, and type of stay were not significant predictors.

For attitude, the model was significant, *R* = 0.33, *R*^2^ = 0.11, *F*(8, 319) = 4.96, *p* < 0.01, accounting for 11% of the variance. Significant predictors included age (*β* = −0.31, *p* = 0.01), academic year (*β* = 0.21, *p* = 0.03), family status (*β* = −0.13, *p* = 0.02), and GPA (*β* = −0.23, *p* = 0.01). Gender, area of residence, family income, and type of stay did not significantly predict attitude scores.

Regarding practice, the model was significant, *R* = 0.38, *R*^2^ = 0.15, *F*(8, 319) = 6.93, *p* < 0.01, explaining 15% of the variance in practice scores. Significant predictors included gender (*β* = 0.13, *p* = 0.02), age (*β* = 0.29, *p* = 0.01), academic year (*β* = −0.32, *p* = 0.01), family income (*β* = 0.20, *p* = 0.01), and type of stay (*β* = −0.18, *p* = 0.01). Family status, area of residence, and GPA were not significant predictors of practice scores. Overall, as shown in Table 5, demographic variables contributed modestly to explaining students’ perceptions, attitudes, and practices regarding ChatGPT, with academic year, age, gender, and GPA emerging as the most consistent predictors across outcomes.

## Discussion

The present study assessed medical students’ perceptions, attitudes, and practices regarding the use of ChatGPT in medical education. Overall, the findings demonstrated a high level of awareness, favorable perception, and positive attitudes toward ChatGPT, accompanied by active but cautious use of this emerging technology. Rather than merely describing usage patterns, these findings provide insight into how generative AI is being integrated into contemporary medical learning environments and how students critically engage with such tools. The vast majority of medical students had previously used ChatGPT, indicating widespread familiarity and exposure of AI-assisted tools among Saudi medical students. This prevalence exceeds that reported in several national (Syed et al., 2023; Alammari et al., 2025; Alharbi et al., 2024) and international studies (Zhang et al., 2024; Abdelhafiz et al., 2025; Ahmed et al., 2025; Hu et al., 2025; Maaß et al., 2024; Ajalo et al., 2025; Moskovich and Rozani, 2025; Fußhöller et al., 2025) where AI literacy and usage remain inconsistent and often limited. The high frequency of use observed in the present study—with more than 86% using ChatGPT often or always, while only 1.08% had never used it. These figures reflect a growing trend of digital engagement and reliance on generative AI in academic learning, consistent with recent global findings that highlight increasing adoption of ChatGPT as a study aid among medical students (Alammari et al., 2025; Abdelhafiz et al., 2025; Hu et al., 2025). This elevated adoption may reflect the increasing academic pressures faced by medical students combined with ChatGPT’s accessibility, real-time feedback, and capacity to simplify complex concepts. However, the predominantly informal and self-initiated nature of its use suggests that institutional integration has not yet kept pace with student engagement. This disconnect highlights a critical need for curricular frameworks that formally address AI-assisted learning while establishing ethical and academic safeguards.

Students’ perception toward ChatGPT were largely positive, with high mean perception scores and strong endorsement of its usefulness for understanding medical concepts and summarizing research literature. These findings are consistent with earlier reports indicating that medical students perceive ChatGPT as a useful adjunct for learning and knowledge consolidation (Alharbi et al., 2024; Hu et al., 2025). Collectively, these findings suggest that students view ChatGPT as a practical tool that support self-directed learning and enhance academic efficacy. However, despite these benefits, a proportion of students expressed reservations about the accuracy and reliability of ChatGPT-generated information. Approximately 40% remained neutral regarding its credibility, and less than half reported full trust in its outputs. This cautious stance is justified, as prior studies have shown that ChatGPT can occasionally produce inaccurate or misleading medical information (Tlili et al., 2023; Wise et al., 2024; Walker et al., 2023; Fatima et al., 2024). The coexistence of perceived usefulness and epistemic caution suggests that students are not blindly adopting AI but are instead negotiating its role as a supplementary rather than authoritative source. This reinforces the importance of embedding AI literacy and critical appraisal skills within medical curricula.

Attitudinal findings further reflected this dual perspective. Students expressed enthusiasm regarding ChatGPT’s role in enhancing learning efficiency and engagement. These results are consistent with previous studies reporting positive emotional responses toward educational chatbots (Ajlouni et al., 2023; Moldt et al., 2023; Acosta-Enriquez et al., 2024). The enjoyment factor associated with interacting with ChatGPT also emerged as motivator for it’s continue use, reflecting its role as novel and engaging educational medium. At the same time, substantial concerns about overreliance, diminished originality, academic misconduct, and ethical implications were evident. More than 60% of respondents acknowledged that excessive dependence on ChatGPT might impair critical thinking and originality — a sentiment echoed in studies by Grassini (2023) and Zhai et al. (2024), which emphasized the potential cognitive risks of AI reliance in higher education. Additionally, concerns regarding plagiarism, misinformation, and data privacy were notable, indicating that while enthusiasm for AI integration is strong, students remain aware of its limitations and potential misuse. Together, these findings indicate that while students welcome AI as a learning enhancement tool, they remain acutely aware of its pedagogical risks — underscoring the necessity of structured ethical governance and instructional oversight.

In terms of practice, students actively incorporated ChatGPT into routine academic tasks, particularly for concept clarification, assignment preparation, and time-efficient information retrieval. A majority agreed that ChatGPT saves times in information retrieval and improves productivity. Notably, over two third of the students stated that they recommend ChatGPT to peers and approximately 75% found it easy to operate without requiring advance technical expertise. These findings demonstrate ChatGPT’s accessibility and convenience as a supportive academic tool, especially for non-technical users. However, student’s cautious approach was reflected in their limited dependence on ChatGPT for verified medical knowledge. Only a minority viewed ChatGPT as a fully reliable source of information, underscoring the need for critical validation of AI outputs. This aligns with prior studies emphasizing the necessity for health professional to verify AI-generated content with authentic academic or clinical sources before application (Ghasemi et al., 2025; Periaysamy et al., 2023; Cong-Lem et al., 2024; Fergus et al., 2023). This balanced utilization pattern reflects an emerging digital competence among medical students—one characterized by technological adoption tempered by professional judgment.

Demographic analyses yielded meaningful insights into adoption dynamics. Analysis of demographic and academic factors revealed significant association with students’ perception, attitude and practice scores. Our findings revealed significant gender differences in both perceptions and practices related to ChatGPT. Male students demonstrated higher perception levels compared with female students, consistent with evidence from prior studies, showing greater awareness and reported use of ChatGPT among male learners (Alammari et al., 2025; Sallam et al., 2023a,b,c; Stöhr et al., 2024). Conversely, female students achieved higher practice scores, reflecting global patterns of stronger engagement with structured educational technologies (Bouzar et al., 2024). This contrast suggests that although male students may exhibit greater initial familiarity or positive perceptions of AI tools, female students may apply these technologies more consistently in academic contexts. Such differences may be attributed to variations in learning approaches, self-regulation, and preferences for organized digital resources, which aligns with previous research demonstrating higher perceived competency among women following the use of ChatGPT-based learning applications (Mashburn et al., 2025). Age also emerged as a significant determinant of students’ engagement with ChatGPT in our study, influencing both attitudes and practical use. Younger students (<21 years) demonstrated more favorable attitudes toward ChatGPT, whereas older students (>21 years) reported higher levels of actual utilization. This pattern is consistent with findings from previous research conducted in Jordan, which identified a positive association between age and frequency of ChatGPT use, indicating that older students are more likely to incorporate the tool into their academic activities (Sallam et al., 2023a,b,c). Such trends may reflect differences in technological familiarity, study organization, and critical engagement with academic resources across academic levels, rather than indicating objectively superior or more effective use of AI-based tools among senior students.

Interestingly, students in the early academic years demonstrated more favorable perceptions, attitudes and practice toward ChatGPT. Our finding echoed with the results of prior research reported that second year students showed favorable attitudes and limited concerned toward chatbots (Stöhr et al., 2024). Our results may be attributed to the adaptability and curiosity characteristic of early learners, as well as their openness to integrating new technologies into their leaning processes. Early year students often face conceptual challenges in medicine and ChatGPT’s ability to simplify complex topics may offer valuable cognitive support. Students from joint family systems in our study demonstrated more favorable perceptions of ChatGPT, which may be attributed to the stronger social support, shared learning environments, and collective encouragement commonly observed in extended households, factors known to positively influence students’ engagement with educational technologies. Conversely, students from nuclear families exhibited more positive attitudes toward ChatGPT, potentially reflecting greater autonomy, self-directed learning tendencies, and openness to adopting digital tools for independent academic work. Furthermore, higher family income was associated with greater practical use of ChatGPT, aligning with existing literature on the digital divide, which consistently shows that students from higher socioeconomic backgrounds have increased access to technological resources, stable internet connectivity, and digital literacy, facilitating more frequent and effective use of educational technologies (Qiu and Ye, 2023; Chen, 2024).

Higher academic achievers demonstrated stronger engagement across all domains, suggesting that academically successful students are more likely to use ChatGPT as supplementary tool rather than a primary source. This association between academic achievement and AI utilization may reflect greater metacognitive awareness and self-regulation among high performing students. This aligns with findings by Youssef et al. (2024), who observed positive associations between AI use and academic performance. However, our results are inconsistent with the study conducted in the UAE, which revealed that students with lower GPA tend to use ChatGPT more frequently as a supportive learning tool (Sallam et al., 2024).

The findings of the present study have an important implication for medical education in Saudi Arabia and beyond. The widespread acceptance of ChatGPT suggest that students are ready for a more formalized integration of AI in learning environment. However, this integration should be accompanied by educational safeguards. Medical educators should emphasize critical thinking, ethical use, and evidence-based validation when employing AI tools. AI literacy workshops, integrated learning modules, and faculty development programs could help ensure that ChatGPT is used as a complementary educational resource rather than a substitute for human reasoning or clinical judgment. In addition, the strong positive responses from early-year students indicate that ChatGPT could serve as a valuable scaffold in foundational medical education, helping students build confidence and comprehension in basic sciences before transitioning to clinical context.

The findings of this study should be interpreted in light of several important limitations. First, the cross-sectional design restricts causal inferences between ChatGPT use and learning outcome. While the data provides valuable insights into current perception, attitude and practice, they do not allow for determining whether ChatGPT use directly enhances or impairs learning performances, critical thinking, or academic integrity. Longitudinal studies or controlled experimental designs are needed to examine the direction and strength of these relationship over time. Second, the study relied on self-reported data, which introduces the potential for recall and social desirability biases. Participants may have overestimated or underestimated their frequency of ChatGPT use or expressed more favorable attitude due to perceived expectation regarding technological competence of academic intelligence. Although anonymity was maintained to minimize such effects, inherent subjectivity in self-assessment remains a methodological constraint. Third, the study was conducted within a single academic institution (King Faisal University) using non-probability convenience sampling, which may limit generalizability of the findings, institutional culture, access to technology, and faculty attitudes toward AI can vary widely across universities and regions. Therefore, the perceptions and behaviors observed in this study reflect only the participating students and should not be generalized to all medical students in Saudi Arabia or internationally. Future multi-institutional research involving more diverse population would enhance the external validity and broader applicability of these findings. Fourth, the rapid evolving nature of generative technologies presents an additional limitation. ChatGPT and similar tools are continuously updated, with new functionalities and improved accuracy emerging regularly. Students’ perceptions, usage pattern, and trust in such technologies are therefore likely to change over time as the tools become more advanced and their applications in education expands. Hence, the current results should be viewed as snapshot of attitudes during early phase of AI integration in medical education rather than a static representation. Lastly, although the questionnaire was designed and validated to capture key constructs such as perception, attitude, and practice, qualitative approaches such as in-depth interviews or focused group discussions could have provided richer contextual understandings of students’ motivations, ethical concerns, and experiences with ChatGPT. Incorporated mixed-method approaches in future studies would yield a more comprehensive understanding of the multifaceted impact of AI tools in medical education.

## Conclusion

This study systematically evaluated medical students’ perceptions, attitudes, and practices regarding ChatGPT use within undergraduate medical students at King Faisal University, Saudi Arabia. The findings revealed ChatGPT has already become an extensively adopted academic tool, characterized by high awareness, frequent usage, and overall positive acceptance among medical students. Rather than functioning as a marginal digital aid, ChatGPT is actively integrated into students’ learning routines for concept clarification, academic writing support, and efficient information retrieval. Importantly, the evaluation revealed a nuanced engagement pattern, wherein students simultaneously recognized ChatGPT’s educational benefits and maintained critical awareness of its limitations. While the tool was perceived as effective in enhancing understanding and study efficiency, students consistently expressed concerns regarding accuracy, overreliance, ethical integrity, and potential impacts on critical thinking. This indicates that ChatGPT is currently viewed as a supplementary learning resource rather than a replacement for authoritative academic or clinical sources. The study further concludes that engagement with ChatGPT is significantly shaped by demographic and academic factors. Younger students, early-year learners, and those with higher academic performance exhibited more favorable perceptions and more frequent use, suggesting that ChatGPT may be particularly effective as a scaffold for foundational medical learning and for students with stronger self-regulated learning strategies. Gender and socioeconomic influences also contributed to variability in adoption patterns. Despite strong individual-level adoption, institutional integration of generative AI within medical curricula remains limited. This misalignment between student practices and formal educational frameworks highlights an urgent need for medical schools to move beyond informal AI usage toward structured, policy-guided implementation. Based on this evaluation, it can be concluded that responsible integration of ChatGPT requires explicit curricular incorporation of AI literacy, ethical governance, and critical appraisal training. Without such frameworks, the educational benefits of ChatGPT risk being undermined by misuse, misinformation, and erosion of higher-order cognitive skills. Overall, this study concludes that ChatGPT holds substantial potential to enhance self-directed learning and academic efficiency in medical education, provided that its use is guided by pedagogical structure and ethical oversight. Proactive institutional engagement with AI technologies will be essential for preparing future physicians who are not only technologically competent but also capable of critically and responsibly navigating digital clinical and educational environments.

## Data Availability

The original contributions presented in the study are included in the article/Supplementary material, further inquiries can be directed to the corresponding author.
