# Supplementary material for: ChatGPT in medical education: a cross-sectional analysis of usage, attitudes, perceptions, and practices among Saudi medical students
Source: Front Artif Intell. 2026 Apr 22;9:1751367. doi: 10.3389/frai.2026.1751367 (PMC13144081; doi:10.3389/frai.2026.1751367)
Supplement: Supplementary file 1 [file Data_Sheet_1.pdf]

Exploring the Attitude, Perception and Practice among Medical Students about ChatGPT

Dear Participant,

I am a researcher at King Faisal University conducting a study titled "Exploring the Attitude, Perception, and Practice of ChatGPT among Medical Students." The purpose of this study is to understand how medical students perceive and use ChatGPT in their academic and clinical learning.

Your participation is voluntary and will involve completing a short questionnaire, which should take approximately 15 minutes. All responses will be anonymous and confidential, and the data will be used solely for research purposes. Your honest responses will greatly contribute to understanding the role of artificial intelligence tools like ChatGPT in medical education.

Thank you for your valuable time and contribution to this study.

Sincerely,

Demographic Questionnaire

Gender	1. Male	2. Female				
Age	1. <21years	2. >21years				
Academic Year	1. Year 1	2. Year 2	3. Year 3	4. Year 4	5. Year 5	
Family Status	Nuclear (small family)	Joint (big Family)				
Area of residence	Urban (city)	Rural (village)				
Family Income	<5000SAR	5001-10000	10001-15000	>15001		
Type of stay	Living with family	University Housing	Sharing Apartment			
GPA in previous Block	4.5 - 5	4 - 4.4	3 - 3.9	2 - 2.9	less than 2	
Have you used ChatGPT before this study? 1. Yes 2. No						
Have you ever used ChatGPT? 1. Never, 2 Sometimes 3. Often 4. Always						

	***Instructions**:* Read each statement and select the response that best describe your perception, attitude and practice. Select the answer that BEST describes you	**Not helpful at all**	**Not helpful**	**Neutral**	**Helpful**	**Extremely helpful**
	**Perceptions towards ChatGPT**					
1	How helpful do you think ChatGPT could be in understanding medical concepts?	1	2	3	4	5
2	How likely do you think it is that ChatGPT could provide accurate medical information?	1	2	3	4	5
3	ChatGPT could be a helpful tool for summarizing research papers	1	2	3	4	5
	**Attitude towards ChatGPT**					
1	I am concerned about the reliability of the information provided by ChatGPT	**Strongly Disagree**	**Disagree**	**Neutral**	**Agree**	**Strongly Agree**
2	I am afraid of relying too much on ChatGPT and not developing my critical thinking skills	1	2	3	4	5
3	I am concerned about the potential security risks of using ChatGPT	1	2	3	4	5
4	I am afraid of becoming too dependent on technology like ChatGPT	1	2	3	4	5
5	I am afraid that using ChatGPT would result in a lack of originality in my university assignments and duties	1	2	3	4	5
6	I am afraid that the use of the ChatGPT would be a violation of academic and university policies	1	2	3	4	5
7	I am concerned about the potential privacy risks that	1	2	3	4	5

	might be associated with using ChatGPT					
8	I am enthusiastic about using technology such as ChatGPT for learning and research.	1	2	3	4	5
9	I believe technology such as ChatGPT is an important tool for academic success.	1	2	3	4	5
10	I think that technology like ChatGPT is attractive and fun to use.	1	2	3	4	5
11	I am always keen to learn about new technologies like ChatGPT.	1	2	3	4	5
12	I trust the opinions of my friends or colleagues about using ChatGPT.	1	2	3	4	5
13	I consider using ChatGPT for help with my studies	1	2	3	4	5
14	I am comfortable with using ChatGPT to generate content for assignments	1	2	3	4	5
15	I am concerned about the potential for misuse of ChatGPT in medical education	1	2	3	4	5
16	I am concerned with the accuracy of information ChatGPT might generate on complex medical topics	1	2	3	4	5
17	I think ChatGPT could be a reliable source for preparing medical exams	1	2	3	4	5
18	It is important for medical schools to establish clear guidelines on the use of AI tools like ChatGPT					
	**Practice of ChatGPT**	Strongly Disagree	Disagree	Neutral	Agree	Strongly Agree
1	ChatGPT helps me to save time when searching for information	1	2	3	4	5
2	For me, ChatGPT is a reliable source of accurate information.	1	2	3	4	5
3	I recommend ChatGPT to my colleagues to facilitate their academic duties	1	2	3	4	5
4	ChatGPT is more useful than other sources of information that I have used previously.	1	2	3	4	5
5	I have used tools or techniques similar to ChatGPT in the past.	1	2	3	4	5
6	I spontaneously find myself using ChatGPT when I need information for my university assignments and duties.	1	2	3	4	5
7	I often use ChatGPT as a source of information in my university assignments and duties.	1	2	3	4	5
8	I think that relying on technology like ChatGPT can disrupt my critical thinking skills.	1	2	3	4	5
9	I appreciate the accuracy and reliability of the information provided by ChatGPT.	1	2	3	4	5
10	I believe that using ChatGPT can save time and effort in my university assignments and duties.	1	2	3	4	5
11	It does not take a long time to learn how to use ChatGPT.	1	2	3	4	5

12	ChatGPT does not require extensive technical knowledge.	1	2	3	4	5
13	I am interested in using more sophisticated AI tools to personalize my learning experience	1	2	3	4	5
